# The ins and outs of the caudal nucleus of the solitary tract: An overview of cellular populations and anatomical connections

**DOI:** 10.1111/jne.13132

**Published:** 2022-05-04

**Authors:** Marie K. Holt

**Affiliations:** ^1^ Centre for Cardiovascular and Metabolic Neuroscience, Department of Neuroscience, Physiology and Pharmacology University College London London UK

**Keywords:** afferent, anatomy, efferent, glucagon‐like peptide‐1, neuropeptides, nucleus of the solitary tract

## Abstract

The body and brain are in constant two‐way communication. Driving this communication is a region in the lower brainstem: the dorsal vagal complex. Within the dorsal vagal complex, the caudal nucleus of the solitary tract (cNTS) is a major first stop for incoming information from the body to the brain carried by the vagus nerve. The anatomy of this region makes it ideally positioned to respond to signals of change in both emotional and bodily states. In turn, the cNTS controls the activity of regions throughout the brain that are involved in the control of both behaviour and physiology. This review is intended to help anyone with an interest in the cNTS. First, I provide an overview of the architecture of the cNTS and outline the wide range of neurotransmitters expressed in subsets of neurons in the cNTS. Next, in detail, I discuss the known inputs and outputs of the cNTS and briefly highlight what is known regarding the neurochemical makeup and function of those connections. Then, I discuss one group of cNTS neurons: glucagon‐like peptide‐1 (GLP‐1)‐expressing neurons. GLP‐1 neurons serve as a good example of a group of cNTS neurons, which receive input from varied sources and have the ability to modulate both behaviour and physiology. Finally, I consider what we might learn about other cNTS neurons from our study of GLP‐1 neurons and why it is important to remember that the manipulation of molecularly defined subsets of cNTS neurons is likely to affect physiology and behaviours beyond those monitored in individual experiments.

## 1 INTRODUCTION

**FIGURE 1:**
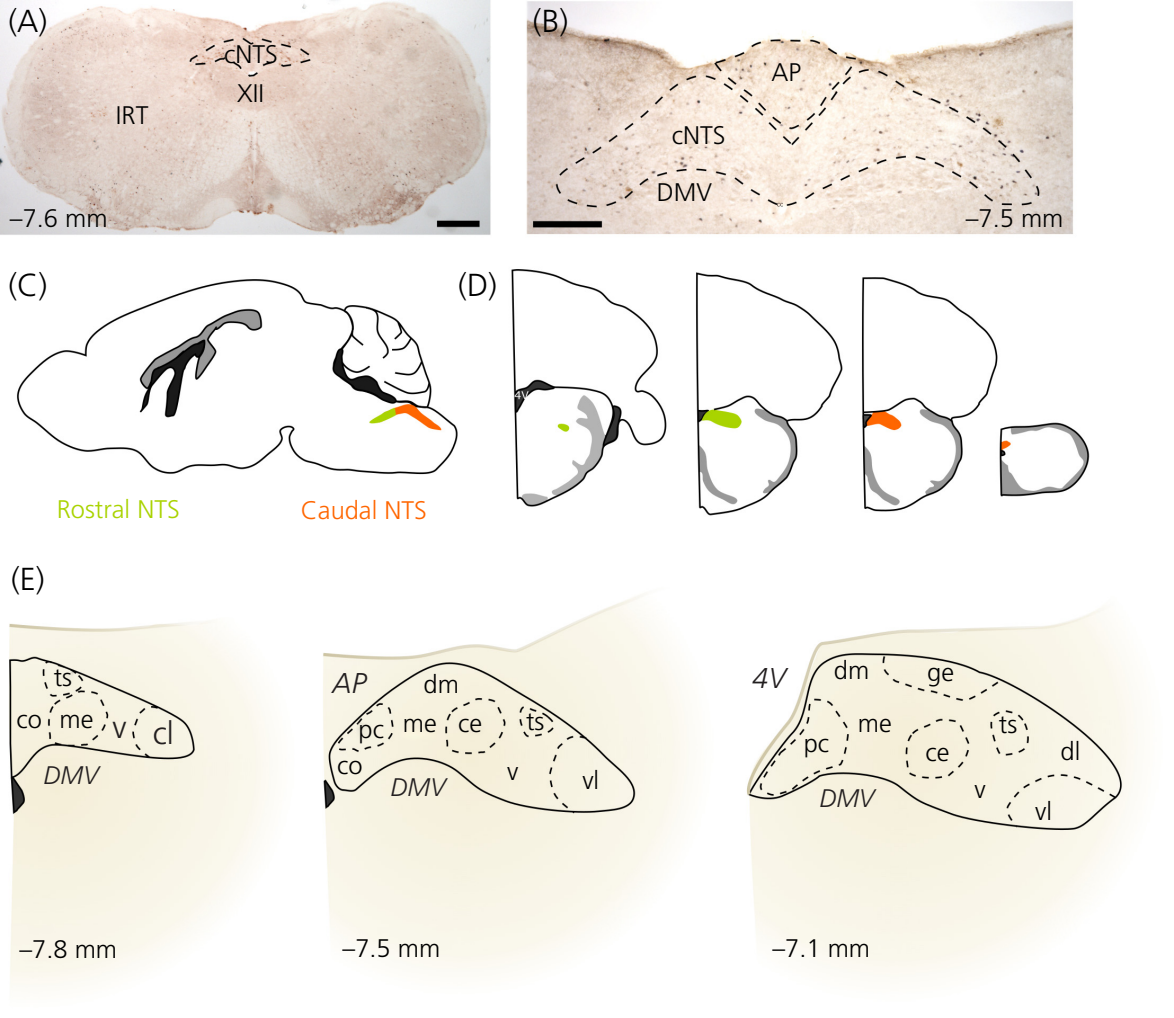
Anatomical organization of the caudal nucleus of the solitary tract (cNTS). (A) Coronal brainstem section containing the cNTS. The hypoglossal nucleus (XII) and the intermediate reticular nucleus (IRT) are indicated as landmarks. Labelled for cFOS (black product) and glucagon‐like peptide‐1 (GLP‐1) (brown product) using immunohistochemistry. Scale bar = 400 μm. (B) Higher magnification of the dorsal vagal complex containing the area postrema (AP), cNTS and dorsal motor nucleus of the vagus (DMV). Labelled for cFOS (black product) and GLP‐1 (brown product) using immunohistochemistry. Scale bar = 200 μm. (C) Schematic of mouse brain with rostral and caudal NTS indicated in green and orange. (D) Schematics of coronal sections through different rostrocaudal levels of the NTS with the rostral part indicated in green and the caudal part indicated in orange. (E) Schematics of cNTS at different rostrocaudal levels (indicated in millimetre from Bregma) with the subnuclei indicated. 4V, fourth ventricle; Ts, solitary tract; co, commissural nucleus; me, medial nucleus; v, ventral nucleus; cl, caudolateral nucleus; pc, parvocellular nucleus; dm, dorsomedial nucleus; ce, central nucleus; vl, ventrolateral medulla; dl, dorsolateral medulla

Our emotional and physical well‐being is carefully monitored by the brain through multimodal pathways. Hormonal input is carried to the brain from the body via the blood, whereas the spinal cord and cranial nerves, including the vagus nerve, carry electrical signals from the periphery to the brain.^1^, ^2^ The afferent (sensory) vagus terminates, among other brainstem nuclei, in the dorsal vagal complex in the lower brainstem. The dorsal vagal complex comprises the nucleus of the solitary tract (NTS), the area postrema (AP) and the dorsal motor nucleus of the vagus (DMV) (Figure 1A,B), with the bulk of the vagal input terminating on second‐order neurons in the caudal part of the NTS (cNTS).^2^ This makes the cNTS anatomically unusual: it receives direct sensory input from the afferent vagus and spinal cord, as well as descending inputs from higher brain regions. This configuration places the cNTS in an ideal position to integrate cognitive information with interoceptive input. Indeed, the cNTS is activated following both interoceptive and psychogenic stimuli.^3^ In turn, the cNTS modulates multiple processes from autonomic outflow to motivated behaviour.^1^, ^4^ Unfortunately, much of the anatomical organization uncovered in the last century is often forgotten in contemporary neuroscience reports, perhaps as a result of the lack of easily accessible, recent overviews. This review is intended to provide exactly that: an overview of the efferent and afferent connections of the NTS, as well as the current state of knowledge regarding the neuropeptidergic cell types residing within the cNTS.

First, I briefly describe the architecture of the cNTS and its resident cell types. Then, I review the anatomical configuration of the inputs and outputs of the cNTS. Finally, as perhaps the best studied peptidergic population of cNTS neurons, glucagon‐like peptide‐1 (GLP‐1)‐expressing neurons will be used as an example of second‐order neurons, which receive substantial vagal sensory input, as well as input from both forebrain and hindbrain regions. This review will not cover the function of the cNTS in detail, and readers are referred to excellent available reviews on the subject.^1^, ^3^, ^4^, ^5^, ^6^

An important note on species is warranted: this review covers only preclinical data, most of which was collected in rodents. Indeed, most of the anatomical data that will be discussed were collected in rat. A small number of tracing and cytoarchitecture studies have been conducted in rabbit,^7^ hamster^8^ and cat,^9^ and, more recently, presumably as a result of the increased popularity of the mouse as an experimental model, reports of the anatomy of the mouse NTS have been added to the literature.^10^, ^11^, ^12^ For clarity and to emphasize the idea that not all species can be reasonably assumed to be anatomically and functionally identical, I will indicate the species that the data were collected from.

## 2 NTS ARCHITECTURE

In rodents, the NTS is traditionally, if somewhat arbitrarily, divided into two parts, sometimes three,^13^ based on their relative rostrocaudal location^10^, ^14^: The rostral or *gustatory* part of the NTS buds dorsolaterally from the spinal trigeminal nucleus to the level of the closure of the fourth ventricle and formation of the AP. It is termed gustatory, because this part of the NTS is the first relay in the central taste neuraxis. The caudal or *visceral* NTS, which receives vagal afferent input originating in the viscera, extends from the opening of the fourth ventricle to the junction between the spinal cord and the lower brainstem (Figure 1C). In coronal sections, the NTS is oval in appearance at more rostral levels (Figure 1D) but takes a triangular shape at the level of the AP, with the DMV situated at its ventral border. At its rostral extreme, the NTS is at its most lateral and gradually moves more medial until it finally surrounds the midline at the very caudal end of the nucleus (Figure 1D).

In rodents, the NTS can be subdivided into a number of subnuclei based on the location, size, shape, density, and staining intensity of neuronal cell bodies following Nissl, silver or Golgi staining. Following this approach, Ganchrow et al.^10^ thoroughly mapped the cytoarchitecture of the mouse NTS and compared this to previous reports in hamster and rat,^10^ which were largely similar. Because Ganchrow et al.^10^ provides such an excellent and exhaustive description of the subnuclear organization of the rodent NTS, this particular aspect of NTS anatomy will not be described in detail here. However, for convenience Figure 1E presents an overview of the subnuclear division of the cNTS.

## 3 CELL TYPES OF THE CNTS

**FIGURE 2:**
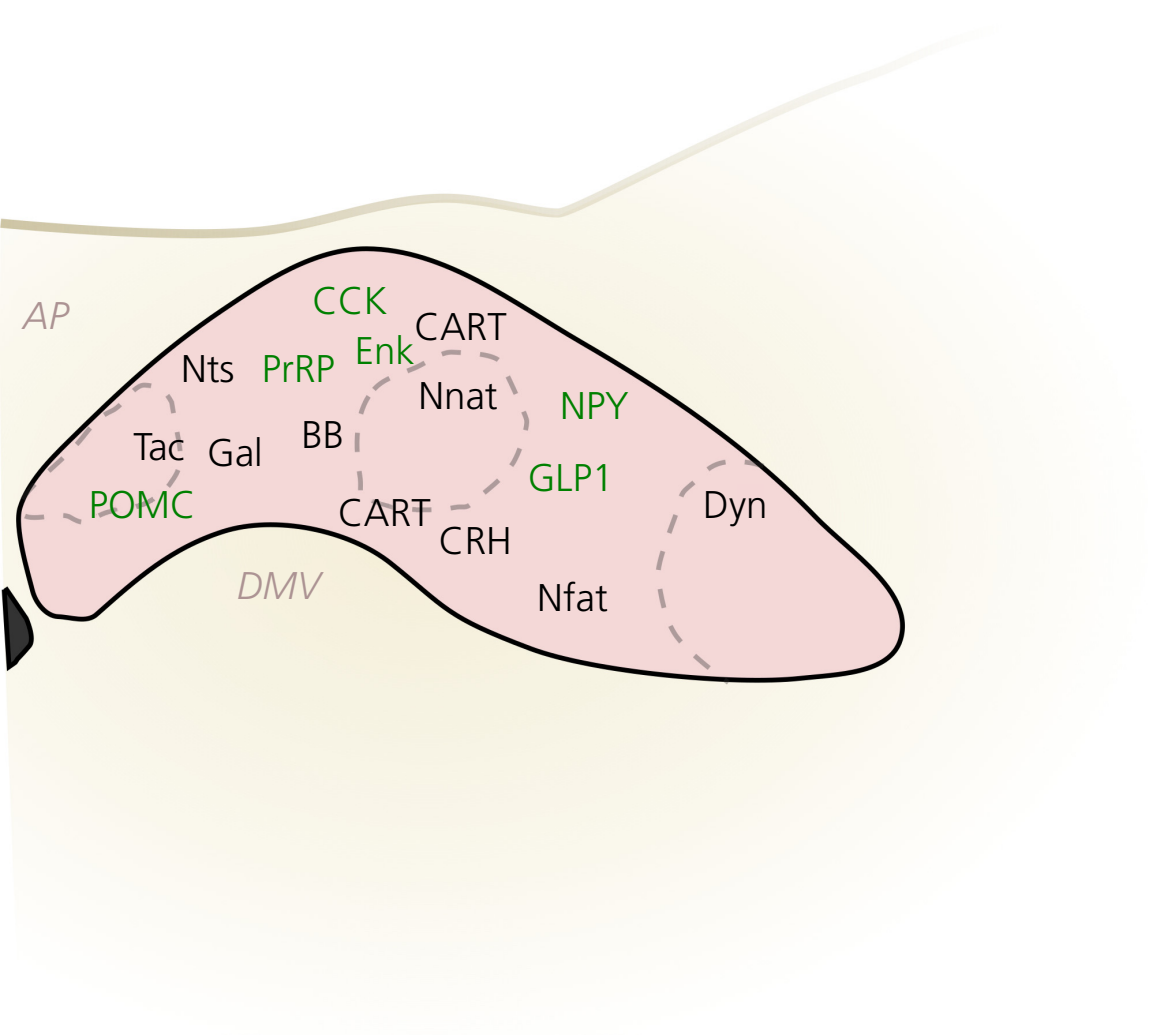
Known peptidergic cell types of the caudal nucleus of the solitary tract (cNTS). Cell types with approximate locations based on published studies referenced in Table 1. Highlighted in green are cell types that have been manipulated chemo‐ or optogenetically to investigate their function as indicated in Table 1. There is conflicting evidence on the location of cocaine‐ and amphetamine‐regulated transcript (CART) neurons, possibly as a result of species differences. For details, see Table 1. Abbreviations are indicated in Table 2

The cNTS is cellularly heterogeneous with a multitude of neuropeptides (Figure 2), small‐molecule neurotransmitters and receptors expressed in distinct or overlapping neuronal populations (Table 1). Not all have been investigated in detail beyond demonstrating their expression in the cNTS and only few have been selectively targeted to study their physiological roles (Figure 2, Table 1). In the last decade, advances in chemo‐ and optogenetic manipulation have made it possible to selectively activate or inhibit cells in an anatomically and genetically defined manner. These advances will not be discussed in detail here, but are highlighted with appropriate references in Figure 2 and Table 1. It is important to note that not all of the listed molecules have been confirmed to be expressed exclusively by neurons. Indeed, astrocytes express both leptin receptors^81^ and GLP‐1 receptors.^82^

**TABLE 1:** Expression of neuropeptides and selected small‐molecular neurotransmitters, intracellular proteins, and receptors in the caudal nucleus of the solitary tract (cNTS)

Neuronal population	Detected in species	Response to cell type‐specific manipulations	References
*Neuropeptides*			
Bombesin‐like peptides (BB)	Mouse, rat	–	15, 16
Cocaine‐ and amphetamine‐regulated transcript (CART)	Mouse, rat	–	17, 18, 19, 20
Cholecystokinin‐8 (CCK)	Mouse, rat	Chemogenetic activation (whole population): food intake↓; conditioned place avoidance; condition taste avoidance Optogenetic activation (fibres in parabrachial nucleus [PBN]): food intake↓; real‐time place avoidance Optogenetic activation (fibres in paraventricular nucleus [PVN]): food intake↓; real‐time place preference	19, 21, 22, 23, 24, 25, 26, 27
Corticotropin‐releasing hormone (CRH)	Mouse, rat	–	26, 28, 29
Dynorphin (Dyn)	Rat	–	27, 30
Enkephalin (Enk)	Rat, mouse	Chemogenetic activation: novel flavour preference↑	27, 30, 31
Galanin (Gal)	Rat, mouse	–	26, 32, 33, 34
Glucagon‐like peptide‐1 (GLP1)	Mouse, rat	Optogenetic activation (whole population and fibres in PVN): food intake↓; Chemogenetic activation: food intake↓; heart rate↑; locomotion↓; glucose production↓; drug reward↓. Chemogenetic inhibition: fast‐refeed↑; stress‐induced hypophagia↓	35, 36, 37, 38, 39, 40, 41, 42, 43, 44
Neuronatin (Nnat)	Rat	–	45
Neuropeptide Y (NPY)	Mouse, rat	Chemogenetic activation: food intake↑	19, 27, 46, 47
Neurotensin (Nts)	Mouse, rat	–	31, 48, 49
Nesfatin‐1 (Nfat)	Mouse, rat	–	19, 50, 51, 52, 53
Proopiomelanocortin (POMC)	Mouse, rat	Optogenetic activation: heart rate↓; breathing↓; Chemogenetic activation: nociception↓; food intake↓; Ablation: food intake↑	17, 54, 55, 56, 57
Prolactin‐releasing peptide (PrRP)	Mouse, rat	Chemogenetic activation: food intake↓ Chemogenetic inhibition: fast‐refeed↑ Ablation: diet‐induced obesity↑	19, 58, 59, 60
Tachykinin/substance P (Tac)	Rat	–	27, 61
*Small molecules*			
GABA	Mouse, rat	Chemogenetic activation: blood glucose↑	62, 63, 64, 65
Glutamate	Mouse, rat	Optogenetic activation: renal and phrenic sympathetic nerve activity↑	66
Noradrenaline (NA)	Mouse, rat	Chemogenetic activation (NET‐Cre; DBH‐Cre): food intake↓ Optogenetic activation (DBH‐cre; fibres in PBN): food intake↓ Optogenetic activation (TH‐cre; fibres in Arc): food intake↑	22, 47, 67
*Intracellular proteins*			
Brain‐derived neurotrophic factor (BDNF)	Mouse, rat	–	19, 68, 69
11β‐hydroxysteroid dehydrogenase 2 (HSD2)	Mouse	Ablation: sodium appetite↓ Chemogenetic activation: sodium appetite↑ Optogenetic activation (fibres in bed nucleus of the stria terminalis): sodium appetite↑	70
Phox2B	Mouse, rat	Chemogenetic activation: breathing↑; food intake↓ Ablation: breathing↓	71, 72
Neuronal nitric oxide synthase (nNOS)	Rat	–	73
*Receptors and transporters*			
Angiotensin‐II receptor (AT2R)	Mouse	Optogenetic activation: systemic blood pressure↑; heart rate↑	74
Glucose transporter 2 (GLUT2)	Mouse	Optogenetic activation: vagal efferent activity↑; blood glucagon↑	75
GLP1 receptor (GLP1R)	Rat, mouse	–	42, 76, 77
Leptin receptor (LEPR)	Mouse, rat	Optogenetic and chemogenetic activation (whole population): breathing↑; food intake↓ Chemogenetic activation (PBN‐projecting): breathing↑	32, 78, 79
Calcitonin receptor (CALCR)	Mouse	Chemogenetic activation: food intake↓ Optogenetic activation (fibres in PBN): food intake↓ Chemogenetic inhibition: food intake↑ Ablation: food intake↑	23
5‐hydroxytryptamine 2C receptor (5‐HT_2C_R)	Mouse, rat	Chemogenetic activation: food intake↓	80

*Note*: Only those with anatomical evidence for expression within neuronal populations resident in the NTS are included. As such, evidence based on physiological or behavioural responses to microinjection of agonists or antagonists into the cNTS has not been included. Also indicated are effects of opto‐ or chemogenetic manipulations of the cellular activity of the particular subpopulation. The list of receptors and transporters is not exhaustive, but highlights a few well‐studied examples.

Abbreviations: DBH, Dopamine β‐hydroxylase; NET, Norepinephrine transporter; TH, Tyrosine hydroxylase.

**TABLE 2:** Abbreviations

IX	9th cranial nerve, glossopharyngeal nerve
X	10th cranial nerve, vagus nerve
AAV	Adeno‐associated virus
AGRP	Agouti‐related peptide
AP	Area postrema
Arc	Arcuate nucleus
Bar	Barrington's nucleus
BB	Bombesin‐like peptides
BST	Bed nucleus of the stria terminalis
CART	Cocaine‐ and amphetamine‐regulated transcript
CCK	Cholecystokinin‐8
CeA	Central amygdala
CRH	Corticotropin‐releasing hormone
CTb	Choleratoxin subunit b
DH	Dorsal horn of the spinal cord
DMH	Dorsomedial hypothalamus
DMV	Dorsal motor nucleus of the vagus
DR	Dorsal raphe
Dyn	Dynorphin
Enk	Enkephalin
Gal	Galanin
Gi	Gigantocellular nucleus
GLP1	Glucagon‐like peptide‐1
IC	Insular cortex
IL	Infralimbic cortex
IML	Intermediolateral column in the spinal cord
IRT	Intermediate reticular formation
IX	Glossopharyngeal nerve
KF	Kölliker‐Fuse nucleus
LC	Locus coeruleus
LH	Lateral hypothalamus
MS	Medial septum
NAc	Nucleus Accumbens
Nfat	Nesfatin‐1
Nnat	Neuronatin
NPY	Neuropeptide Y
Nts	Neurotensin
NTS	Nucleus of the solitary tract
cNTS	Caudal nucleus of the solitary tract
OT	Oxytocin
OVLT	Vascular organ of lamina terminalis
PAG	Periaqueductal grey
PBN	Parabrachial nucleus
POMC	Proopiomelanocortin
PPY	Parapyramidal region
PrL	Prelimbic cortex
PrRP	Prolactin‐releasing peptide
PSTh	Parasubthalamic nucleus
PVN	Paraventricular nucleus of the hypothalamus
PVT	Paraventricular nucleus of the thalamus
RLi	Linear raphe nucleus
RMg	Raphe magnus
ROb	Raphe obscurus
RPa	Raphe pallidus
SFO	Subfornical organ
SI	Substantia innominata
Sp5	Spinal trigeminal nucleus
Tac	Tachykinin/substance P
VH	Ventral horn of the spinal cord
VLM	Ventrolateral medulla
VMH	Ventromedial hypothalamus
VTA	Ventral tegmental area
ZI	Zona incerta

### 3.1 NTS glia in the modulation of information flow in the NTS

In addition to neurons, astrocytes contribute heavily to the function of the cNTS.^5^ Based on immunolabelling for glial‐fibrillary protein (GFAP), astrocytes appear to be more densely packed in the rat NTS^82^, ^83^ than in the mouse NTS,^76^, ^84^ although, to this author's knowledge, a direct, quantitative comparison has not been made. Of note, in both species, the densest expression of GFAP is found in the border region between the AP and the cNTS,^83^, ^84^, ^85^ where astrocytes may regulate transport of molecules across the border and thus modulate the flow of information from the blood into the NTS.^81^, ^85^ In addition to forming a selectively permeable diffusion barrier between the AP and the NTS, astrocytes in the rat NTS form part of tripartite synapses, specialized synaptic arrangements consisting of a synaptic cleft containing the pre‐ and postsynaptic terminals covered by astrocytic processes.^86^ Interestingly, NTS astrocytes are activated in response to vagal stimulation in rats and NTS gliotransmission modulates the synaptic transmission of second‐order NTS neurons in rats.^87^

In addition to astrocytes, microglia and oligodendrocytes express neurotransmitter receptors and microglia in the NTS are altered in response to varied stimuli, including removal of vagal input^83^ (rat), obesity^88^ (rat), and hypoxia^89^ (mouse). A detailed discussion of NTS glial function is beyond the scope of this review, and readers are referred to a recent comprehensive review on the subject.^5^, ^90^, ^91^

## 4 AFFERENT CONNECTIONS OF THE CNTS

**FIGURE 3:**
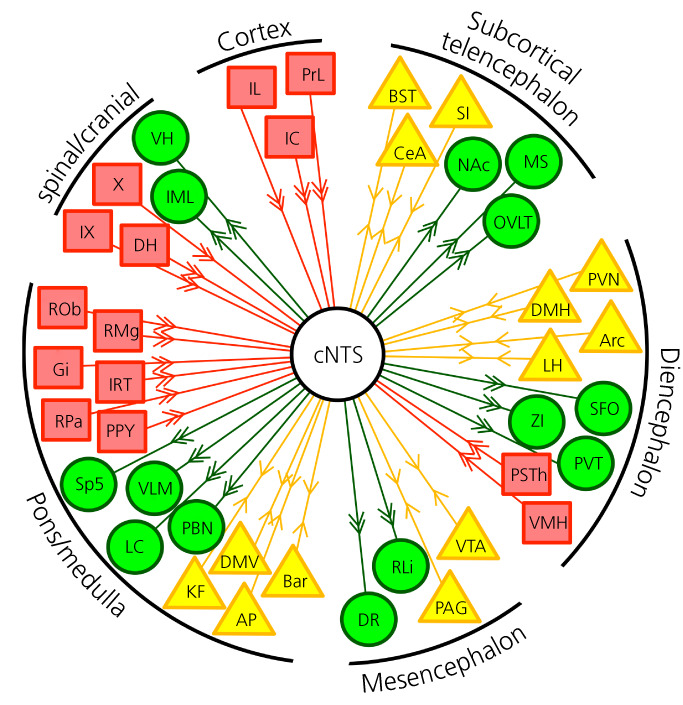
Efferent and afferent inputs to the caudal nucleus of the solitary tract (cNTS). Direct inputs to the cNTS are indicated in red boxes. Projection targets are indicated in green circles. Areas with bidirectional connections are in yellow triangles. For abbreviations, see Table 2

Input to the cNTS arises from widespread regions in the brain, as well as peripheral sites (Figure 3), comprising an anatomical organization that is reminiscent of the significant variety of physiological and psychogenic stimuli, which modulate the activity of the NTS.^92^ These inputs have been reported predominantly in rats, although studies using mice, rabbits and cats are also included here. Studies mapping the monosynaptic input to the NTS take one of two forms: (1) injection of an anterograde tracer (typically phaseolus vulgaris leucoagglutinin [PHA‐L], 10,000 MW biotin dextran amine [BDA], or adeno‐associated virus [AAV]) from a hypothesized source of input to the NTS and subsequent validation of the presence of labelled axons in the NTS or (2) injection of a retrograde tracer (typically wheatgerm agglutinin‐horseradish peroxidase [WGA‐HRP], choleratoxin subunit B [CTb], fluorogold, or a retrograde AAV) into the NTS and subsequent mapping of retrogradely labelled brain regions. In addition, a few studies have mapped inputs to molecular defined subpopulations of cNTS neurons using cell‐type specific monosynaptic and polysynaptic retrograde tracing.^12^, ^93^

### 4.1 Sensory inputs to the cNTS

The cNTS is directly sensitive to blood‐borne signals, including changes in glucose,^94^ leptin^46^ and angiotensin II.^95^ In addition, visceral sensory information is transmitted via the afferent vagus and glossopharyngeal nerves to the cNTS where glutamatergic terminals synapse onto second‐order neurons.^1^, ^96^, ^97^ Peripheral chemo‐ and baroreceptors sense changes in blood pressure, as well as the pH, temperature and composition of the arterial blood.^6^ This information is relayed by the afferent glossopharyngeal and vagus nerves to the NTS.^6^ In addition to the continuous monitoring of cardiovascular and pulmonary function, the cNTS receives information from the abdominal viscera via the afferent vagus nerve.^1^, ^2^, ^97^ Vagal sensory terminals in the cNTS appear to be topographically organized, such that input from the heart and lungs terminate in different subnuclei.^98^, ^99^ These vagal sensory neurons express a range of receptors and signalling molecules, recently mapped in detail by Bai et al.^100^ using RNA sequencing data. Ultimately, this transcriptomic and anatomical specificity facilitates appropriate information flow from the viscera to the cNTS.^1^, ^2^ Additional sensory input to the NTS arises from the dorsal horn of the spinal cord,^12^, ^101^ perhaps relaying signals of tactile and nociceptive stimuli, although very little is known about these spinal inputs. These monosynaptic inputs from peripheral organs makes the NTS the primary brain region to receive and process rapid, neurochemical information regarding the internal environment of the body. Indeed, the cNTS is robustly activated in response to interoceptive stimuli.^4^, ^6^, ^92^ Interestingly, however, the cNTS also receives widespread central inputs and is engaged following psychogenic stressors, suggesting that the cNTS is also sensitive to information regarding emotional states.^3^, ^12^, ^102^

### 4.2 Central inputs to the cNTS

Central inputs to the NTS, which appear to be similar in rats and mouse,^11^ are depicted in Figure 3 alongside efferent outputs. Below, I describe, in some detail, our current state of knowledge of the central inputs to the NTS. Of note, few studies have been able to limit the injection of retrograde tracers to the cNTS without significant leakage to more rostral areas or to the AP and DMV. This limitation makes it difficult to conclude with certainty which regions provide input to the cNTS specifically. In addition, many retrograde tracers, including the widely used CTb, are taken up by fibres of passage. In the case of the NTS, this could mean any descending projections to the spinal cord not terminating in the NTS may take up and transport CTb.^103^ For a few brain regions, those limitations have been addressed by combining anterograde and retrograde tracing.

#### 4.2.1 Telencephalic inputs

The insular, prelimbic and infralimbic cortices all provide significant bilateral input to the NTS in mice^11^, ^12^ and rats.^11^, ^104^, ^105^ In rats, infralimbic neurons directly synapse onto catecholaminergic neurons in the NTS.^106^ These descending inputs appear to mediate cortical modulation of sympathetic and parasympathetic activity^107^, ^108^ and, as such, may represent a functional link between emotional processing and autonomic outflow.

Subcortically, several regions of the extended amygdala innervate the NTS, including the central amygdala and bed nucleus of the stria terminalis of rat,^11^, ^14^, ^105^ mouse^11^, ^12^ and rabbit.^109^ Interestingly, all of these extended amygdala inputs appear to be exclusively ipsilateral.^11^ Most input from the central amygdala arises from the medial subdivision in both rats^11^, ^110^ and mice,^12^ although the lateral subnucleus also provides some synaptic input.^12^ In the rat cNTS, central amygdala inputs terminate mostly in the medial and dorsomedial subnuclei and not only are predominantly GABAergic,^14^ but also may release a range of neuropeptides as co‐transmitters, including nociceptin in mice^111^ and somatostatin, neurotensin and vasoactive intestinal polypeptide in rats.^112^

#### 4.2.2 Diencephalic inputs

Arguably the densest central input to the NTS arises from the paraventricular nucleus of the hypothalamus (PVN), evidenced in rats^11^, ^105^, ^113^ and mice.^11^, ^12^ This input is bilateral,^11^ primarily originates in the more caudal parts of the PVN^114^ (rat) and appears to represent a distinct parvocellular population, which does not overlap with neuroendocrine magnocellular PVN neurons in mice^11^ and rats.^115^ In rats, 60% of NTS‐projecting PVN neurons express the stress neuropeptide corticotropin‐releasing hormone (CRH),^114^ and PVN CRH neurons project directly to the NTS in mice.^116^ Evidence from rats suggests that a much smaller population (6%–10%) of NTS‐projecting PVN neurons express oxytocin,^114^, ^117^ PVN axons in the NTS express oxytocin,^118^ and electrical stimulation of the PVN leads to release of oxytocin into the dorsal vagal complex.^119^ In mice, PVN oxytocin cells do not provide significant direct synaptic input to the cNTS, although oxytocinergic fibres are clearly visible in the NTS of mice.^120^ Finally, a subset of NTS‐projecting PVN neurons express the melanocortin 4 receptor.^121^ Removal of this descending input from the PVN leads to the development of obesity,^122^ although data from mice suggests this pathway has no effect on ad libitum feeding.^123^ One possibility is that stress, a powerful stimulus to suppress eating in rodents, activates NTS‐projecting PVN neurons in mice,^12^ which in turn mediate stress‐induced activation of cNTS neurons, including those that express catecholamines in rats.^124^

Other hypothalamic inputs include the arcuate nucleus, the dorsomedial hypothalamus, and the lateral hypothalamus in both mice^11^, ^12^ and rats.^11^, ^105^ In addition, neurons in the ventromedial hypothalamus may innervate the NTS in mice,^125^ although not every comprehensive study reported input from the dorsomedial and ventromedial hypothalamus in mouse.^11^, ^12^ Interestingly, descending input from the arcuate nucleus does not appear to arise from agouti‐related peptide or pro‐opiomelanocortin (POMC) neurons in mouse,^93^ while in rat, evidence suggest a small population of POMC neurons do project to the dorsal vagal complex.^126^

Finally, the parasubthalamic nucleus provides heavy, unilateral input to the NTS in mice^11^, ^12^ and rats,^11^ a pathway that may mediate fear‐induced changes in autonomic outflow,^127^ although studies of this particular nucleus are scarce. Indeed, the phenotype of these NTS‐projecting parasubthalamic neurons remains unknown, but may include tachykinin‐expressing,^128^ CRH‐expressing^129^ and/or glutamatergic neurons.^127^

#### 4.2.3 Mesencephalic and hindbrain inputs

The periaqueductal grey, Edinger–Westphal nucleus, parabrachial nucleus, Kölliker‐Fuse nucleus and Barrington's nucleus all provide direct input to the NTS in rats and mice.^11^, ^12^, ^130^, ^131^ Parabrachial input appears to mainly arise from glutamatergic, non‐calcitonin‐gene related peptide neurons in mice,^132^ whereas tachykinin‐expressing neurons in the periaqueductal grey may be the source of input to the NTS in rats.^133^, ^134^ We recently found that NTS‐projecting Barrington's nucleus neurons are activated in response to acute restraint stress in mice and express the stress neuropeptide CRH,^12^ supporting the idea that the NTS is engaged following psychogenic stimuli.

Finally, multiple lower brainstem regions provide input to the NTS in the mouse and rat, including the raphe obscurus, the raphe magnus, the reticular nucleus, the parapyramidal regions, the gigantocellular nucleus^11^, ^12^ and the DMV^135^ (rats). Interestingly, input from the raphe magnus nucleus appears to partly mediate activation of cNTS neurons in response to interoceptive stressors, including LiCl,^136^ and 5‐hydroxytryptamine signalling in the NTS (arising from either vagal afferents or the raphe nuclei) is an important modulator of central control of autonomic outflow^137^ and feeding behaviour.^138^

## 5 EFFERENT CONNECTIONS

Far from being a simple reflex station, which relays information from the afferent to the efferent vagus, the cNTS sends projections throughout the subcortical central nervous system, including to many autonomic control centres.^139^ I was unable to find a comprehensive, analysis of the efferent connections of the *mouse* NTS based on injection of an anterograde tracer. However, some retrograde tracing in mouse has been reported for individual target regions and the findings from those studies will be included here when relevant. In addition, the anterograde mapping of specific subpopulations of cNTS neurons in transgenic mouse models does provide us with some idea of the outputs of the cNTS in mouse. One example of this type of anterograde tracing was carried out by Shi et al.^140^ who mapped long‐range GABAergic inputs from the NTS.

### 5.1 Circumventricular organs

Neurons in the cNTS send projections to a number of sensory circumventricular organs: the AP,^141^ the subfornical organ^142^ and the vascular organ of laminar terminalis.^143^ NTS input to the subfornical organ is inhibitory and may relay signals from peripheral baroreceptors in the rat.^144^

### 5.2 Telencephalic projections

Notably, there is no evidence that the cortex or any of the hippocampal regions receive monosynaptic input from NTS neurons. Subcortically, the entire extended amygdala receives input from NTS neurons: The bed nucleus of the stria terminalis, the nucleus accumbens, the medial septum, the substantia innominata and the central amygdala are all synaptic targets of cNTS neurons in the rat.^31^, ^143^, ^145^, ^146^ At least a subset of NTS inputs to the bed nucleus of the stria terminalis in mice are GABAergic,^140^ suggesting that this pathway is partly inhibitory, although other NTS cell types are known to project to these regions as well, including GLP‐1 neurons in mouse^35^ and rat^143^ and catecholaminergic neurons in rat,^146^ but not NTS POMC neurons in mouse.^93^

### 5.3 Diencephalic projections

Diencephalic targets include multiple regions in the hypothalamus. The PVN is a particularly densely innervated region in rats^139^, ^143^, ^147^ and mice,^140^ and the input is at least partly made up of GLP‐1^35^, ^143^ (mouse and rat), catecholaminergic^143^ (rat), GABAergic^140^ (mouse) and POMC fibres (mouse).^93^ Other hypothalamic targets include the dorsomedial hypothalamus, the lateral hypothalamus and the arcuate nucleus.^143^, ^147^ These inputs are at least partly made up of GLP‐1 and catecholaminergic projections in the rat^143^ and GABAergic^140^ and GLP‐1^35^ in the mouse. Additional diencephalic targets include the paraventricular thalamus and zona incerta in rat.^143^, ^147^

### 5.4 Mesencephalic and pontine projections

In the midbrain, the ventral tegmental area, the dorsal raphe and the periaqueductal grey all receive input from the NTS in the rat.^143^ Further caudal, the Kölliker‐Fuse nucleus, parabrachial nucleus, locus coeruleus and Barrington's nucleus are targets of NTS efferents in rats.^143^ Efferents to the parabrachial nucleus are assumed to drive suppression in appetite,^148^ and, in the mouse, include input from POMC,^93^ GLP‐1,^35^ CCK^21^ and noradrenergic neurons.^22^ In the very caudal pons, the rostroventrolateral medulla^9^ (cat) and DMV^9^, ^149^ (cat and rat) make up a subset of the brain‐wide autonomic control centres, which receive dense projections from the NTS. Finally, the neighbouring AP receives light input from the cNTS in the cat.^141^

### 5.5 Spinal connections

In cats, the NTS projects to the thoracic ventral horn, the intermediolateral spinal column and phrenic motor neurons in the cervical spinal cord, suggesting some direct modulation of sympathetic outflow through spinal projections.^9^ In mice, the trigeminal spinal nucleus and the principle sensory nucleus of the trigeminal receive dense input from GABAergic NTS neurons.^140^

## 6 GLP‐1 NEURONS: A WIDELY‐PROJECTING SECOND‐ORDER POPULATION WITH DIVERSE MODULATORY ROLES

Although it is essential that we understand the anatomical connections of the cNTS as a whole, this nucleus is transcriptionally heterogenous and individual subpopulations of neurons are unlikely to serve identical functions or receive identical inputs (Table 1). In recent decades transgenic mouse models and viral gene transfer tools have facilitated investigations of the anatomy and function of anatomically and molecularly defined cell populations. Transgenic mice expressing Cre recombinase (Cre) under cell‐type specific promoters allow selective targeting using cre‐dependent viruses. As an example, *‐Cre* transgenic mice express Cre under the control of the glucagon (*Gcg*) promoter.^150^ Because GLP‐1 is also expressed under the *Gcg* promoter, this results in Cre expression selectively in GLP‐1 neurons in the lower brainstem and olfactory bulb (in addition to glucagon‐ and GLP‐1‐expressing cells in the periphery).^150^ Injection of a cre‐dependent AAV into the dorsal vagal complex of these mice leads to expression of a desired transgene, often a chemogenetic receptor, an anterograde tracer or channelrhodopsin‐2 for functional and/or anatomical investigations.

Using these techniques, GLP‐1 neurons in the caudal brainstem are now relatively well understood, anatomically, cellularly and functionally. Here, I provide a very brief overview of their function and anatomy. Interested readers are referred to recent reviews on the subject for a more comprehension discussion.^92^, ^102^, ^151^

### 6.1 GLP‐1 neurons: Distribution and innervation

Within the cNTS, GLP‐1 is expressed predominantly in glutamatergic neurons in the commissural, medial and ventral subnuclei in mice and rats,^35^, ^36^ and GLP‐1 neurons innervate widespread autonomic control centres, as well as nuclei involved in modulation of motivated behaviour in rats^143^ and mice,^35^ including the rostroventrolateral medulla, the PVN, dorsomedial hypothalamus and the bed nucleus of the stria terminalis. Whether specialized subpopulations of GLP‐1 neurons innervate distinct targets is still unknown, although classic tracing studies, demonstrating that 30%–40% of GLP‐1 neurons innervate distinct targets, would suggest some level of collateralization.^152^, ^153^ Use of retrogradely transported AAVs to target GLP‐1 neurons based on their projection target could reveal the extent of their collateralization. If anatomically distinct subpopulations exist, is it likely these are also functionally distinct? A previous finding indicating that stimulation of GLP‐1 receptors in the central amygdala increases anxiety‐like behaviour, whereas injection into the PVN decreases food intake without affecting anxiety‐like behaviour, would suggest at least some separation of functions.^154^ However, it does not necessarily follow that specialized subpopulations exist. GLP‐1 neurons could simply modulate a range of diverse processes simultaneously. Future studies selectively manipulating subsets of GLP‐1 neurons based on their innervation targets should address these questions.

### 6.2 Monosynaptic inputs to GLP‐1 neurons

Until recently, mapping the monosynaptic inputs to molecularly defined cell populations was not possible. The recent development of Envelope‐A pseudotyped, G‐deleted Rabies virus (EnvA‐ΔG‐RABV) encoding GFP or mCherry represented a significant step forward.^155^ In combination with Cre‐expressing transgenic mice or rats, this genetically modified rabies virus is efficient, exclusively retrograde, strictly monosynaptic and cell‐type specific.^155^ Using such a EnvA‐ΔG‐RABV we recently mapped the monosynaptic inputs to GLP‐1 neurons in the mouse cNTS^12^ and found that GLP‐1 neurons receive dense monosynaptic input from many of the same regions that provide input to the cNTS as a whole. Notable exceptions included cortical regions, the arcuate nucleus, the ventral tegmental area and the linear raphe nucleus.^12^ We also identified polysynaptic inputs, including the hippocampal formation, the arcuate nucleus and the paraventricular thalamus.^12^

### 6.3 Does anatomy predict function?

By mapping the inputs and outputs of subpopulations of NTS neurons we improve our understanding of their physiological functions with the interesting question often being: how do subpopulations differ anatomically? Unfortunately, we still have only few whole‐brain maps of molecularly defined, anatomically distinct subpopulations of NTS neurons: POMC and GLP‐1 neurons.^12^, ^93^ Based on these maps, we now know that the monosynaptic inputs to cNTS GLP‐1 and POMC neurons are similar with a few notable exceptions: There appears to be some, albeit limited, monosynaptic input from cortical regions to POMC neurons in the mouse,^93^ and, although the lateral subnucleus of the central amygdala provides the majority of the input the GLP‐1 neurons,^12^ POMC neurons receive their input from the medial subnucleus.^93^ Considering that the lateral and medial subnuclei of the central amygdala have distinct inputs, outputs and expression profiles,^110^, ^156^ this could suggest POMC and GLP‐1 neurons form part of distinct brain circuits. Mapping these circuits is one step, although understanding their role in the modulation of behaviour and physiology is hampered by our difficulty in specifically manipulating subsets of neurons that provide direct synaptic inputs to molecularly defined cell types. The limiting factor has been the toxicity of rabies virus, leading to cell death within weeks of infection.^157^ Further improvements in the toxicity of rabies viruses have been reported and may allow specific populations of input neurons to be manipulated for behavioural testing.^157^

Regarding efferent connections, cNTS GLP‐1 neuron projections appear to be significantly more widespread than those of NTS POMC neurons in the mouse.^93^ Although GLP‐1 neurons innervate multiple regions in the extended amygdala and hypothalamus,^35^, ^143^ the only forebrain regions to receive input from cNTS POMC neurons are the PVN, the PSTh, and the medial subnucleus of the central amygdala.^93^ It will be interesting to determine whether these differences in inputs and outputs are matched by differences in function. Although both populations decrease food intake, an important functional difference appears to be their effect on heart rate: optogenetic activation of cNTS POMC neurons leads to a *decrease* in heart rate,^54^ but chemogenetic activation of GLP‐1 neurons *increases* heart rate.^37^ Interestingly, both GLP‐1 and POMC neurons are activated by solitary tract stimulation and CCK,^158^, ^159^, ^160^ suggesting at least some overlap in the stimuli that engage them.

### 6.4 The importance of remembering the bigger picture in the study of single subpopulations of cNTS neurons

Peptidergic cNTS neurons, and perhaps GLP‐1 neurons in particular,^92^, ^102^ are exquisitely well‐positioned to integrate interoceptive or psychogenic signals. It is their anatomical configuration that enables this integration of multimodal signals of physical and mental well‐being. In turn, GLP‐1 neurons have the ability to impact truly varied processes both autonomic and behavioural (Table 1) and they are activated by both interoceptive (LiCl, gastric distension, large volume of food intake) and psychogenic stimuli (stress).^92^ It is unknown whether individual GLP‐1 neurons drive one, some, or all of these functions (i.e., whether functional subpopulations of GLP‐1 neurons exist). Given that GLP‐1 neurons likely collateralize significantly (see above on distribution and innervation GLP‐1 neurons) we might speculate that individual GLP‐1 neurons drive multiple processes simultaneously. Importantly, it is likely that GLP‐1 neurons are not the only cNTS neurons with a very broad anatomical and functional profile (Table 1) and, when investigating the function of these other populations, we should keep in mind that they are not unlikely to modulate multiple downstream targets and, as a result, multiple physiological and behavioural processes simultaneously, as discussed for GLP‐1 neurons above. Examples of this ability to modulate multiple processes are provided in Table 1. Subpopulations of neurons do not work in isolation in the living organism and their artificial activation through chemo‐ or optogenetics is likely to have impact beyond the single output measured in most experiments. The cNTS is perhaps particularly sensitive to this as a result of its position as a link between sensory and emotional inputs, as well as its ability to modulate both behaviour and physiology.

## 7 CONCLUSIONS AND FUTURE DIRECTIONS

The afferent inputs to the NTS make it ideally suited to respond to both psychogenic and interoceptive stimuli, whereas its efferent connections facilitate widespread modulation of autonomic function and motivated behaviour. However, the specific circuits and cell types contributing to the functions of the NTS are still not fully understood. Future studies should take advantage of recently developed retrograde AAVs and the targeting of ChR2‐expressing terminals to selectively manipulate neurons, based on not only their neurochemical phenotype, but also their projection targets. These circuit‐ and cell type‐specific studies will, in combination with previously published classic knife‐cut and toxin studies, provide new insights into the functions of subpopulations of cNTS neurons.

## CONFLICTS OF INTEREST

The author declares that he has no conflicts of interests.

8

### PEER REVIEW

The peer review history for this article is available at https://publons.com/publon/10.1111/jne.13132.

## Data Availability

Data sharing is not applicable to this review because no new data were created or analyzed.
